# Recent Advances in Chitosan and its Derivatives in Cancer Treatment

**DOI:** 10.3389/fphar.2022.888740

**Published:** 2022-04-28

**Authors:** Jingxian Ding, Yonghong Guo

**Affiliations:** ^1^ Department of Radiation Oncology, The Breast Cancer Institute, The Third Hospital of Nanchang, Nanchang, China; ^2^ Department of Radiation Oncology, The Fourth Affiliated Hospital of Nanchang University, Nanchang, China

**Keywords:** cancer treatment, anticancer drugs, drug delivery system, chitosan, polysaccharides

## Abstract

Cancer has become a main public health issue globally. The conventional treatment measures for cancer include surgery, radiotherapy and chemotherapy. Among the various available treatment measures, chemotherapy is still one of the most important treatments for most cancer patients. However, chemotherapy for most cancers still faces many problems associated with a lot of adverse effects, which limit its therapeutic potency, low survival quality and discount cancer prognosis. In order to decrease these side effects and improve treatment effectiveness and patient’s compliance, more targeted treatments are needed. Sustainable and controlled deliveries of drugs with controllable toxicities are expected to address these hurdles. Chitosan is the second most abundant natural polysaccharide, which has excellent biocompatibility and notable antitumor activity. Its biodegradability, biocompatibility, biodistribution, nontoxicity and immunogenicity free have made chitosan become a widely used polymer in the pharmacology, especially in oncotherapy. Here, we make a brief review of the main achievements in chitosan and its derivatives in pharmacology with a special focus on their agents delivery applications, immunomodulation, signal pathway modulation and antitumor activity to highlight their role in cancer treatment. Despite a large number of successful studies, the commercialization of chitosan copolymers is still a big challenge. The further development of polymerization technology may satisfy the unmet medical needs.

## 1 Introduction

Cancer has been becoming a life-threatening disease and a major public health problem all over the world, which is one of the leading causes of death. It is reported that the probability of people with invasive cancer is about 40 and 25% in the United States and China, respectively (Chen et al., 2016; Pan et al., 2017; Cao et al., 2020; Siegel et al., 2021; Sung et al., 2021). Cancer treatment depends on the type of cancer, the stage at diagnosis and the patient’s tolerance. The available anticancer therapies include surgery, chemotherapy, radiotherapy, and immunotherapy and so on (Miller et al., 2019). Among the various available therapies, chemotherapy is still one of the most important treatment measures for most cancer patients, which is regarded as one of the main treatment approaches to prevent cancer cell proliferation. However, the active chemicals used to treat cancer usually do not distinguish cancer cells from healthy cells because both are exposed to cytotoxic chemotherapeutic drugs, which results in a high rate of severe adverse reactions and limits its therapeutic effect (Grosso and De-Paz, 2021). To improve therapeutic potency and minimize the side effects, more targeted therapies are highly needed (Perez-Herrero and Fernandez-Medarde, 2015). Under this circumstance, researchers around the world have explored a variety of new nanocarriers and targeted modification systems to overcome these shortcomings and improve the therapeutic outcomes (Ghaz-Jahanian et al., 2015; Bai et al., 2021; Shakeran et al., 2021; Dubey et al., 2022). Because nanotechnology provides a suitable means for the targeted and time-controlled delivery of drugs and other bioactive agents, it has been widely studied in drug delivery and has potential application prospects in cancer treatment (Sabra et al., 2017; George et al., 2019; Ion et al., 2021; Maspes et al., 2021; Sohrabi and Packirisamy, 2021; Li et al., 2022). Drug delivery systems (DDSs) refer to the methods of delivering drugs to the targeted tissues, organs, cells or subcellular organs through various drug carriers for controlling drug release and absorption, so as to improve the pharmacological activity, overcome the limited solubility, low bioavailability, poor biological distribution and lack of selectivity, or to minimize the adverse effects (Li et al., 2019). Polymer based DDSs may potentially improve current disease treatment because they can pass through a variety of biological barriers to overcome the shortcomings of insoluble drugs, increase half-life and suppress the side effects of toxic drugs. Among them, chitosan-based nanocarriers were probably the most interesting, flexible and bio-compatible systems (Lin et al., 2017; Zhao et al., 2017; Naskar et al., 2019; Monteiro et al., 2021).

As a natural polysaccharide, chitosan can be found in some types of seafood, such as shrimp, crab and crayfish, and the content is particularly high in their shell. The main properties of chitosan are biocompatibility, biodegradability and non-toxicity (Herdiana et al., 2021; Zhu et al., 2021; Zivarpour et al., 2021). Chitosan has a high concentration of reactive free protonable amino groups along the chitosan backbone, and due to the protonation of amino groups, it presents higher solubility in acid environment. These groups can be chemically modified in a variety of chemical reactions to enhance its solubility, biocompatibility and targeting activity (de Sousa Victor et al., 2020; Ding et al., 2020). Consequently, chitosan has the potential to use in a variety of fields (Aranaz et al., 2021). The positive charge of chitosan allowing for non-covalent interactions with biological tissues has been used in drug delivery, which may assist to overcome the inadequacy of the existing chemotherapy. Some kinds of therapeutic agents conjugated with chitin or chitosan derivatives have displayed wonderful anticancer potency with less adverse effects than the original drugs due to targeted distribution into the cancer and sustainable release (Jia et al., 2014; López-Barrera et al., 2021; Tang et al., 2021). Normally, there are two traits in cancer tissue: acidic pH and the reductive environment, which have been heavily studied as internal trigger for the drug release in smart DDSs (Friedl and Alexander, 2011).

Additionally, chitosan may accumulate in the tumor site, initiate the polarization of M1 macrophages and transform the immunosuppressive tumor microenvironment to immunosupportive state, thus exerting an antitumor effect and promoting the efficacy of cancer immunotherapy (Ding et al., 2020; Zeng et al., 2021). What’s more, chitosan can also activate innate immune responses to exert its anticancer effect (Chakrabarti et al., 2014; Li et al., 2018; Yang et al., 2019a; Wu et al., 2019; Chen et al., 2020; Van Hees et al., 2020; Liang et al., 2021; Lima et al., 2021).

Last but not least, Chitosan itself may also inhibit tumor cell growth, tumor induced angiogenesis and tumor metastasis. Consequently, chitosan and its derivatives may have high performances in cancer treatment fields. There are varieties of publications in this quite fantastic field over last decade. Herein, in this review, based on the current tumor epidemiology and treatment dilemma, we gathered the state-of-the-art publications on chitosan to show its application in anticancer drug-delivery, innate immune stimulation, cancer cell inhibition and signal modulation, providing more information about its characteristics, chemical modifications, and applications in cancer treatment. Finally, we briefly prospected the future trends and challenges of chitosan nanoparticles in cancer treatment.

## 2 Cancer and its Epidemiology

**FIGURE 1:**
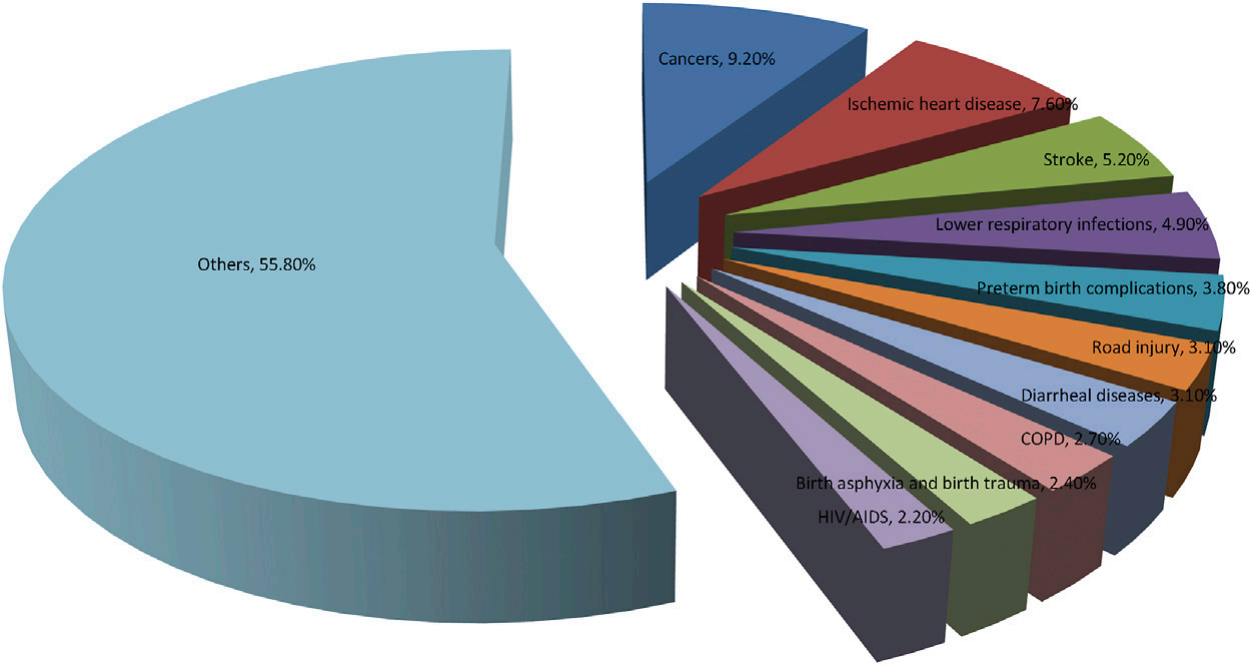
World Health Organization (WHO) Global Health Estimates 2019 on the 10 leading causes of disease burden, estimated as cause-specific Disability Adjusted Life Years (DALYs). Cancer has become the leading causes of disease burden by cause-specific disability adjusted life years.

Cancer is not only the main cause of death, but also a vital obstacle to longevity all over the world. Based on estimates from the World Health Organization (WHO) in 2019, cancer has become the most common cause of death before the age of 70 years in most countries (Sung et al., 2021). From here we see that cancer has become a major public health issue to be solved urgently worldwide. WHO newly updates the 10 most common causes of disease burden by cause-specific disability adjusted life years (DALYs), which is shown in Figure 1.

**FIGURE 2:**
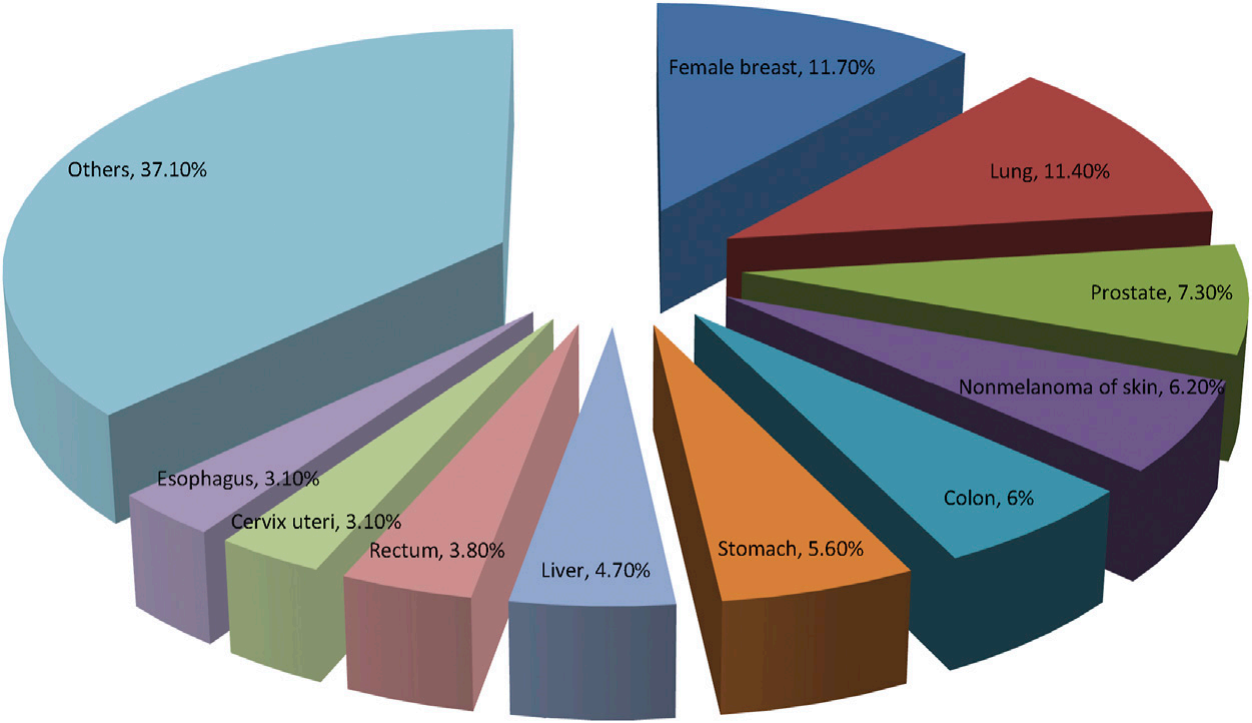
The prevalence of the top 10 most common cancers according to the GLOBOCAN 2020. Breast cancer has replaced lung cancer as the most common cancer worldwide.

In general, cancer has become the highest clinical, social, and economic burden among all human diseases. The economic burden associated with cancer has a profound impact on the health and non-health outcomes of cancer survivors. American Cancer Society and International Agency for Research on Cancer provide recent information on 10 most frequent types of cancers worldwide in Figure 2.

**FIGURE 3:**
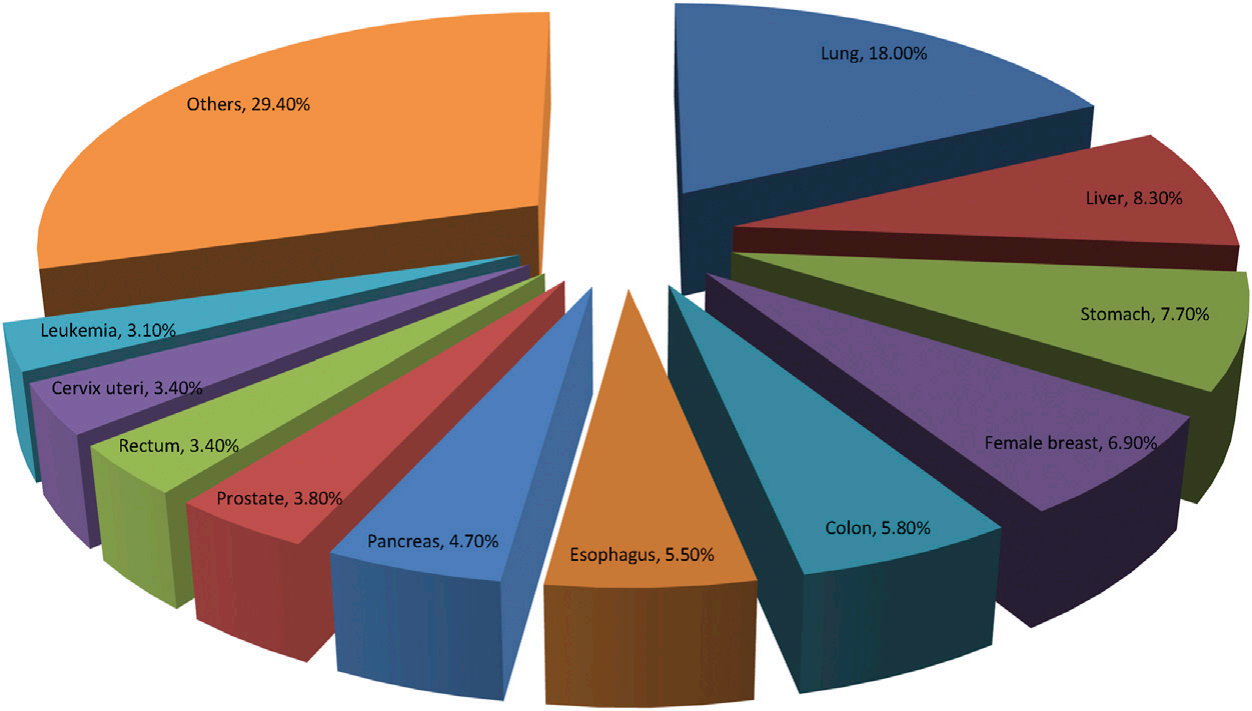
Distribution of top 10 deaths of Cancers according to the GLOBOCAN 2020. Lung cancer remains the most common cause of cancer related-mortality all over the world.

GLOBOCAN 2020 has summarized the most recent changes of cancer burden all over the world. It is reported that there were nearly 20 million new cases of cancer and about 10 million deaths from cancer in 2020 globally. The top three common cancers were female breast cancer, lung cancer and prostate cancer worldwide. While, lung cancer, liver cancer and stomach cancer are the three of cancer death in general population, lung and breast cancers are most common causes of cancer related-mortality in men and women, respectively (Sung et al., 2021). Figure 3 illustrates the top 10 mortality cancer worldwide. Relatively, China has a lower cancer incidence but higher cancer mortality compared with the developed countries, such as United States and United Kingdom. The high mortality rate in China may be due to the different cancer spectrum, the low diagnosis rate of early cancer and the inconsistent clinical cancer treatment strategies implemented among different regions. Overcoming cancer has become a big global challenge.

## 3 Cancer Treatment: Prospects and Dilemmas

Cancer is one of the most deadly and life-threatening diseases in the world, causing about 13% of deaths every year. Traditional cancer treatment strategies, such as surgery, chemotherapy, radiotherapy, hyperthermia and immunotherapy, have been developed for cancer therapy. However, these treatment measures often inevitably have their shortcomings, such as low inhibition efficiency and no targeting to cancer cells, which eventually lead to treatment failure. For example, surgical procedures may affect various pathophysiological processes, thereby promoting cancer cell proliferation and tumor recurrence. What’s more, surgical manipulation may increase the dissemination of cancer cells to the circulation and induce local or systemic immunosuppressive responses, which finally increase the chance of colonization in target organs (Narmani and Jafari, 2021). Chemotherapy is the most commonly used measure in the existing cancer treatments. However, chemotherapeutic drugs may lead to serious problems, such as detrimental side effects, drug resistance and heavy financial burden. Similarly, both chemotherapy and radiotherapy cannot differentiate cancer cells from normal healthy cells in patients, and thus both are damaged by treatment. Although significant progress has been made in cancer treatment, the therapeutic effects and overall survival for patients with cancer are still unsatisfactory, especially for patients with advanced cancer. In order to reduce the rate of adverse events, improve therapeutic efficacy and patient’s compliance, more targeted therapies are urgently needed.

Cancer immunotherapy is under the spotlight recently, which is completely different from traditional cancer treatment. It is now considered to be a potentially effective way to conquer cancer. Cancer immunotherapy regulates the immune system rather than focusing on the cancer cells (Esfahani et al., 2020; Mu et al., 2020). However, immunotherapy has encountered a number of challenges in some kinds of cancer, including resistance of immune checkpoint inhibitors, weak immunogenicity of therapeutic vaccines, serious immune related side effects, and off-target effects and so on. Chemoimmunotherapy is a combination of chemotherapy and immunotherapy, which provides synergistic effect for improving anticancer potency. It is a promising cancer treatment method, with the advantages of cooperating two kinds of treatment mechanism (Nowak and Lesterhuis, 2014). The very recent studies demonstrated that nanoparticles could remodel immunosuppressive tumor microenvironment, holding great promise for chemoimmunotherapy (Xu et al., 2022). Moreover, nanobased delivery systems have exhibited excellent properties, such as targeted drug delivery, tumor microenvironment modulation, targeted and time-controlled release of conjugate drugs, so as to improve the pharmacokinetics and stability of drugs *in vivo*.

In the journey of exploring new cancer treatment measures, the development of nanobased drugs is expected to become a promising method to overcome the current shortcomings of traditional chemotherapy drugs (Nazir et al., 2014). So far, many nanobased anticancer drugs have been developed and extensively studied both *in vitro* and *in vivo*, some of which have been under clinical trials in the past few decades. The antitumor effect of nanocarriers is achieved by delivering drug selectively to the tumor cells. Based on the source, drug carriers can be classified into two types: chemosynthetic and natural. Natural biopolymers such as chitosan, collagen, cellulose, and fibrin, are vastly investigated in the field of pharmacology owing to their unique characteristics. In particular, chitosan based nanoparticles have drawn considerable attention as chemotherapeutic drug delivery carriers because of their easy accessibility, terrific stability, toxicity free, and modification friendly (Zhang et al., 2019).

## 4 Chitosan: Source, Structure, Physicochemical Properties and Application in Oncotherapies

### 4.1 Source, Structures and Physicochemical Properties

#### 4.1.1 Source of Chitosan

Chitosan, a unique alkaline polysaccharide in nature, is found in the invertebrate shelves (Farhadihosseinabadi et al., 2019). Shrimp, crabs, lobster, crayfish and oyster are the most common origins for chitosan preparation (Marsili et al., 2021). However, chitosan for industrial scale application is normally derived from chitin by a process of deacetylation. Chitin, one of the richest natural polymers, is a polymer of N-acetyl-D-glucosamine. When deacetylation is carried out, the repeating units in the chitin normally do not contain β-1,4-D-glucosamine functional groups, and this chitin is called chitosan. The mole fraction of the N-acetylated repeating units is regarded as the degree of acetylation (DA), on the contrary, the percentage of the repeating units of β-1,4-D-glucosamine in the polysaccharides is defined as the degree of deacetylation (DD). Although there are no universal agreement on the cut-off values for DD between chitin and chitosan, the DD values of most commercial chitosan are between 70 and 90% (Younes and Rinaudo, 2015; Shahbaz, 2020; Kou et al., 2021).

#### 4.1.2 Structures of Chitosan

The molecular structure of chitosan was first resolved comprising of β-1,4-D-glucosamine and N-acetyl-D-glucosamine units in 1950 (Zhang et al., 2019). The molecular weight (MW), DD, degree and site of substitutions carry heavy weight on physiochemical properties of chitosan and its derivatives as well as the antitumor potency of chitosan-based nanoparticles. The average MW of commercially produced chitosan varies from 3,800 to more than 300,000 Da (Bhavsar et al., 2017; Adhikari and Yadav, 2018). Chitosan can be classified as low MW chitosan, medium MW, and high MW chitosan depending on the DD. Low MW chitosan has been proved to be more effective in size reduction, solubility enhancement and nanocrystal formulations’ stability, thereby being considered as a promising nanocarrier to formulate oral sustainable release drugs to improve the bioavailability (Naqvi and Moerschbacher, 2017).

Generally, the chitosan with MW up to 10 kDa is known as chitosan oligosaccharide, which obtains from degradation of chitosan, and exhibits a lot of exciting molecular weight-dependent biological traits, especially antitumor activities (Bonin et al., 2020). Therefore, extensive studies have been conducted to convert chitosan to chitosan oligosaccharides with specific molecular weight in order to find more effective nanocarriers with both economy and environment friendly properties. Tremendous efforts have been made to produce chitosan oligosaccharides in the past decades. Generally, all of these methods come from chemical or enzymatic approaches. Based on chemical approach, acidic hydrolysis has been widely applied for chitosan oligosaccharides production (Salehinik et al., 2021).

#### 4.1.3 Physicochemical Properties of Chitosan

Chitosan is generally considered as a safe and biocompatible polymer material. Based on most studies, chitosan has no toxicity or little toxicity, so it is widely considered to be a safe biomaterial. Chitosan shows several biomedical properties, such as antimicrobial, antitumor and hemostatic activities, which is dependent on the chitosan MW and DD (Park et al., 2011). Molecular weight is a vital factor influencing the physical and chemical properties of chitosan based nanoparticles. Normally, high MW increases both the stability of chitosan-based complexes and circulation time of nanoparticles in blood stream but delays their dissociation and subsequent effect in cells, which subsequently guarantees the high tumor selectivity, while lower MW has the opposite effects.

The DD represents free amino groups in the chitosan structure which can be determined by different methods, also carries out a vital role in physical, chemical and medical properties (Rodrigues et al., 2021). It determines the positive charge density and the ability to bind with DNA/siRNA. Higher DD helps to improve the efficiency of transfection by escaping from the endolysosomal compartment. According to the percentage of DD, chitosan can be categorized into low DD of chitosan, medium DD of chitosan, high DD of chitosan and ultra-high DD of chitosan four different forms, which with DD values between 55 and 70%, 70 and 85%, 85 and 95% and between 95 and 100%. Generally, high DD will make nanoparticles with high surface charge density, thus enhancing cell uptake and antitumor effect. In traditional method, the DD of chitosan obtained is nearly 80%, while ultra-high DD chitosan is still difficult to obtain for medical application scale.

What’s more, the degree and site of substitutions grafted onto chitosan also affects the physical, biochemical traits and the antitumor potency. The abundance of deoxycholic acid and hydrophobic group affects nanoparticle size, entrapment efficiency and drug loading content significantly. The site of carboxymethylation on chitosan can influence the stability and deformability of nanoparticles, which is also strongly correlated with the antitumor activity and cellular uptake of carboxymethyl chitosan conjugates. Furthermore, the abundance of protonated-NH2 groups on the chitosan structure determines its solubility in acid medium, since its pKa value is about 6.5. However, the solubility window of chitosan can be changed by use of hydrogen bond disruptors such as urea or guanidine hydrochloride. In fact, a wide range of solubility can be achieved through the chemical or physical destruction of hydrogen bonds. Though the characteristics of chitosan and its derivatives have great influence on the biochemical effect of chitosan particles, it should be noted that these factors do not work alone, but have a comprehensive impact on the chitosan conjugates. Thus, to ensure the superior *in vivo* tumor targeting efficiency, solubility, stability and deformability of nanoparticles should be balanced. Selecting appropriate chitosan as nanocarriers needs to comprehensively consider the relationship between the above factors and payload properties, preparation methods of nanoparticles, targeted diseases and so on (Narayanan et al., 2014).

In addition, from a technical point of view, the viscosity of polymer is another very important parameter because high viscosity solutions are difficult to manage. Viscometry is a powerful tool for determining the molecular weight of chitosan because it is a simple and rapid method, although it is not the only method (Wang and Xu, 1994). Glycosidic bonds and acetylamine groups can also be regarded as functional groups, which allow a large number of modifications to produce polymers with new properties and behaviors. One of the most important properties of chitosan is its cationic property. Because electrostatic interaction enhances the adhesion to the negatively charged mucosal surface, chitosan plays an advantage as an ideal drug carrier, which improves the internalization of drugs into target cells (Kurakula et al., 2015). On the other hand, the cationic nature of chitosan also makes it possible to prevent anionic rich nucleic acids from degradation by nucleases in serum, improving the efficiency of gene therapy.

In order to improve the properties of chitosan or introduce new functions or properties to chitosan, many chitosan derivatives have been produced, among which hydrophobically modified ethylene glycol chitosan is one of the most commonly used derivatives for the preparation of self-assembled nanoparticles. The abundant amino and hydroxyl groups on the chitosan skeleton represent more target parts of chemical modification, which improves water solubility and endow chitosan with some new functions, such as targeted and environmentally sensitive drug release, and enhances therapeutic effect and minimize side effects. The most common chitosan derivatives used as drug carriers include thiochitosan, trimethyl chitosan, carboxymethyl chitosan, ethylene glycol chitosan and so on (Hu et al., 2020; Grosso and De-Paz, 2021; Li et al., 2021).

### 4.2 Applications of Chitosan in Oncotherapies

#### 4.2.1 Chitosan-Based Delivery System

Tremendous efforts have been made to maintain long-term therapeutic levels of drug concentration in the targeted site, administration time span, and decrease side effects for disease treatment. The search for novel controlled drug release systems is closely related to the establishment of more effective treatment approaches that can be administered more safely and with few side effects. Chitosan with good biocompatibility and biodegradability is one of the most functional natural biopolymers widely used in the pharmaceutical field. The properties of chitosan make it a promising nanocarrier, which has been used to deliver variously therapeutic agents such as chemotherapeutic drugs, peptides/proteins, vaccines, DNA/siRNA, and so on (Shikhi-Abadi and Irani, 2021).

##### 4.2.1.1 Chemotherapeutic Drugs Delivery

Various nanocarriers have been used to deliver chemotherapeutic drugs to tumor sites. These nanocarriers normally can evade the immune surveillance system, achieve target selectivity, gain access into the interior of cancerous cells, evade endosomal entrapment and release the drugs in a sustainable manner (Wang et al., 2021). Chitosan has attracted the attention as a promising candidate for the drug carrier and has been vastly exploited in the last decade because of its innate favorable properties (Dousti et al., 2021; Du Q et al., 2021). Basically, there are three categories of chitosan nanoparticles namely self-assembled nanoparticle, ionic cross-linked nanoparticle and polyelectrolyte complex, depending on different preparing methods (Zhang et al., 2019).

Classical chemotherapeutic drugs, such as doxorubicin, paclitaxel, cisplatin and so on, have made tremendous contributions to cancer treatment in last nearly half a century. However, these drugs also bring too many severe side effects, which restrict the intensively clinical use to cure cancer. A large number of studies have been conducted to solve these severe side effects of chemotherapeutic drugs. DDSs are introduced to targeted and sustained release of drugs in a controllable manner to minimize the adverse effects. Chitosan-based nanoparticle is a useful DDS, which can encapsulate and deliver various antitumor drugs to specific tumor tissues (Fathi et al., 2018; Pornpitchanarong et al., 2020; Ryu et al., 2020).

In addition to being encapsulated, chemotherapeutic drugs can also be covalently bound to hydrophilic polymers and self-assembled to form nanoparticles, which are called polymer drug conjugates. The conjugates of some anticancer drugs with chitosan and its derivatives selectively accumulate in tumors and prolong retention time in the blood circulation, thus show excellent anticancer effects with much milder adverse effects than that of the original drug due to cancer site-specific distribution and sustained release characteristics. Based on the cationic properties of chitosan, ionic cross-linked chitosan nanoparticles were prepared by electrostatic interaction, in which the amino group on the main chain interacts with polyanionic cross-linking agents such as tripolyphosphate (TPP), CaCl2, Na2SO4 and so on (Babu and Ramesh, 2017; Nemati et al., 2021). The process of ionically cross-linking requires more simple and mild preparation conditions with no toxic reagents, which is completely different from the chemical cross-linking. Furthermore, the biochemical properties of ionically cross-linking nanoparticles, such as size and surface charge, can be easily modified by adjustment of processing parameters, which changes drug encapsulation potency and time controlled release profile. However, the ionic cross-linked chitosan based particles are weak. It is reported that ionic cross-linked chitosan nanoparticles can improve tumor targeting of some ligands, such as folic acid, herceptin antibody and glycyrrhetinic acid (Tian et al., 2010; Arya et al., 2011; Hefnawy et al., 2020). Table 1 lists a few examples of chitosan based chemotherapeutic agents nanoparticles and their application in oncotherapies.

**TABLE 1:** A few examples of chitosan based chemotherapeutic drugs nanoparticles and their application in oncotherapies.

Drugs	Application	Results	References
Docetaxel	Lung cancer	Ameliorated the immunosuppressive microenvironment to promote the antitumor effects	Zhu et al. (2021)
MMC	Hepatocellular carcinoma	Achieved high accumulation at the tumor site and more efficiently suppress the tumor cells growth	Jia et al. (2014)
Gemcitabine	Breast cancer	Minimized the side effects, improving therapeutic potency	Karuppaiah et al. (2021)
Cisplatin	Ovarian cancer	Showed controlled release of cisplatin, and enhanced therapeutic efficacy	Khan et al. (2020)
MTX	Cervical cancer	Targeted tumor extracellular drug release	Naghibi et al. (2017)
Norcantharidin	Hepatocellular carcinoma	Prolonged retention time in blood circulation and reduced biodistribution in heart and kidney tissues	Chi et al. (2019)
Dox	Breast cancer	Selective and sustainable release of free doxorubicin site-specific to the breast tumor microenvironment	Helmi et al. (2021)
5-Fu	Breast cancer	Released the drug in a controlled manner	Zavareh et al. (2020)
Ara-C	Leukemia	Displayed a good pH-dependent release in an acid tumor environment	Chen et al. (2021)
Camptothecin	Ovarian cancer	Maximized the anticancer and antimetastatic effects and reduce its toxicity	Zhou et al. (2010)

MMC, mitomycin C; MTX, methotrexate; Dox, doxorubicin; 5-Fu, 5-fluorouracil; Ara-C, cytarabine.

##### 4.2.1.2 Chitosan-Based Systems as Therapeutic Genes Carriers

Gene-based therapy has become an indispensable part of comprehensive tumor therapy, which is achieved by delivering exogenous nucleic acids (DNA/siRNA) that can regulate gene expression of tumor cells (Senapati et al., 2019). Small interfering RNA (siRNA) system has been used for down-regulation of targeted gene and subsequent inhibition of cancer progression, which is a promising strategy in cancer treatment (Ashrafizadeh et al., 2021; Ferdows et al., 2022). SiRNA is a category of double stranded oligonucleotides containing 20–25 pairs of nucleotides, which is used to knock down targeted messenger RNA (mRNA) to induce gene silencing. However, its off-targeting property and degradation by enzymes in serum and the extracellular matrix prevent it application in cancer therapy. What’s more, therapeutic nucleic acids can be degraded by endonucleases in serum and extracellular matrix easily. Therapeutic nucleic acids are easily degraded by endonucleases in serum and extracellular matrix. Therefore, the development of effective vectors is the vital to successful gene therapy. Chitosan has attracted large attention as a promising polymer vector for delivery gene products because of its minimal toxicity, low immunogenicity, terrific biodegradability and its cationic property. The negatively charged gene products can bind to protonated cationic amino groups on chitosan backbone via electrostatic interaction, which shield them from being degraded by nucleases. Hence, the delivery of siRNA in chitosan-based nanocarriers is an important way to enhance its efficacy in gene silencing. However, due to the instability *in vivo* and insufficient cell release of natural chitosan, its transfection efficiency is relatively low. Therefore, it is most necessary to chemically modify natural chitosan to develop a more powerful chitosan based delivery system (Ragelle et al., 2013). Moreover, chitosan can also provide a platform for the co-delivery of siRNA and antitumor agents. In the past several years, a large number of studies have reported a lot of different chitosan-based delivery systems with terrific antitumor effects for a variety of therapeutic gene silencing nucleic acids. These systems achieved successful *in vivo* delivery of therapeutic genes siRNA with desirable tumor specificity and transfection efficiency, which suggested that adjusting the binding strength between vector and siRNA is very important to improve both transfection efficiency and antitumor activity. For instance, applying stimulation responsive chitosan based nanoparticle to co-deliver sgVEGFR2/Cas9 plasmid and Ca2+ channel siRNA demonstrated markedly increasing tumor targeting and therapeutic efficacy both *in vitro* and *in vivo* (Zhao et al., 2020; Li et al., 2021). Table 2 lists a few examples of chitosan based gene delivery nanoparticles and their application in oncotherapies.

**TABLE 2:** A few examples of chitosan based therapeutic genes nanoparticles and their application in oncotherapies over the last decades.

DNA/siRNA	Application	Results	References
PD-L1-siRNA	Breast cancer and melanoma	Showed a significant inhibitory effect on proliferation and migration *in vitro*, angiogenesis and tumor growth *in vivo*	Nikkhoo et al. (2020)
RRM2-siRNA	Ovarian cancer	Effectively inhibited tumor growth in nude mice models of subcutaneous transplantation of tumor cells	Xue et al. (2019)
Snail-siRNA	Prostate cancer	Inhibit the proliferation and migration of PC-3 cells in vivro	Afkham et al. (2018)
Survivin-siRNA	Breast cancer	Significantly inhibited tumor cell growth and enhanced cellular uptake nanoparticles to reduce the growth of xenograft tumors	Sun et al. (2016)
HMGA2-siRNA	Hepatocellular carcinoma	More effectively induced tumor cell death and significantly reduced the expressions of HMGA2	Siahmansouri et al. (2016)
STAT3-siRNA	Lewis lung cancer	Resulting in a significant reduction in STAT3 expression and successfully transferring macrophages from M2 phenotype to M1 phenotype	Senel and Ozturk, (2019)
IGF-1R siRNA	Non-small cell lung cancer	Significantly decreased the motility of A549 cells and inhibited the expression of MMP9, VEGF and STAT3	Shali et al. (2018)
BCL2-siRNA	Non-small cell lung cancer	Inhibited tumor growth effectively by down regulating BCL2	Zhang et al. (2019)
MDR1-siRNA	Cervical cancer	Prevented siRNA from degrading and produced a chemosensitized phenotype of the multidrug resistant cancer cells	Heidari et al. (2021)
Ang2-siRNA	Melanoma	Efficiently inhibited Ang-2 expression, tumor angiogenesis, and induced the melanoma cells apoptosis through the mitochondrial apoptotic pathway	Shan et al. (2020)

PD-L1, programmed death ligand 1; RRM2, ribonucleotide reductase regulatory subunit M2; HMGA2, high mobility group AT-hook 2; STAT3, signal transducer and activator of transcription 3; IGF-1R, insulin like growth factor 1 receptor; MDR1, multidrug resistance gene; Ang2, angiopoietin 2

##### 4.2.1.3 Chitosan-Based Nanosystems as Carriers of Other Therapeutic Agents

Photodynamic therapy (PDT) is one of the most promising research fields in oncotherapy, which combines a light source with a photosensitizing agent preferably located within the tumor to destroy cancer. Photosensitizers are activated by light and transfer energy to molecular oxygen to produce reactive oxygen species (ROS), resulting in the death of targeted cells (Sun et al., 2022). PDT rapidly increases in cancer treatment due to its minimal toxicity. However, selectively deliver water-insoluble photosensitizers into target tissues is still a big challenge, more intelligent chitosan-based nanoparticles are urgently needed. It is reported that the pH-dependent conjugates exhibited photo toxicity against tumor cells. Furthermore, chlorin e6 (Ce6) and glycol chitosan self-assembled into nanoparticles attenuated its cytotoxicity but improved its therapeutic efficacy by preventing the nanoparticles from serum absorption and prolonged the blood circulation time (Ding et al., 2018). These chitosan-based nanosystems have been successfully applied to photodynamic therapy and photothermal therapy for breast cancer with minimal side effects (Liu et al., 2018).

Chitosan and its derivatives as drug carriers are also applied for cancer immunotherapy (Yang et al., 2021). Interleukin-12 (IL-12) is a heterodimeric pleiotropic cytokine with immunomodulatory activities, which has been intensively studied either as an antitumor agent or a vaccine adjuvant. However, the severe systematic toxicities hinder its clinical applications. Nevertheless, IL-12 conjugates with chitosan nanoparticles have marked effect on inhibiting growth of liver metastatic tumors of colorectal cancer with no obvious toxicity (Xu et al., 2012).

Moreover, it is reported that a chitosan coated copper sulfide (CuS) nanoparticles incorporated with immunoadjuvants which exerted antitumor activity. The chitosan coated nanoparticles are decomposed after laser excitation and then reassemble into chitosan CpG complex, which activates host anti-tumor immunity eventually. The chitosan coated copper sulfide (CuS) nanoparticles therapy shows a more effective effect than immunotherapy or photothermal therapy alone, resulting in synergistic effects against both primary treated and distant untreated tumors (Guo et al., 2014; Niu et al., 2021). Table 3 lists a few examples of chitosan based other therapeutic agents nanoparticles and their application in cancer treatment.

**TABLE 3:** A few examples of chitosan based other therapeutic agents nanoparticles and their application in oncotherapies over the last decades.

Agents	Application	Results	References
FA-CS	Breast cancer	Improved delivering capacity to cancer cells through ligand-receptor dependent and independent cellular engulfment	Nemati et al. (2021)
GA-CS	Hepatocellular carcinoma	Through enhancing intracellular delivery and uptake to improve both antitcancer efficacy and safety	Hefnawy et al. (2020)
HER2-Gem-CS	Pancreatic cancer	Showed superior anti-proliferative activity and enhanced S-phase arrest due to higher cellular binding and prolonged intracellular retention	Arya et al. (2011)
CNPs-Ce6	Lung cancer cells	Attenuated its cytotoxicity but improved its therapeutic efficacy by preventing serum absorption and prolonging the blood circulation time	Ding et al. (2018)
CS-TPP/IL-12	Colorectal cancer	Attenuated the toxicity of IL-12 and inhibited tumor metastasis by inducing NK cells and T cells infiltration	Xu et al. (2012)
HMSNs-CS-DOX- CuS	Breast cancer	Site-specific release of DOX under the tumor microenvironment, preventing release into circulation	Niu et al. (2021)
Erlotinib- CNPs	Lung cancer cells	Released erlotinib slowly in comparison to the marketed tablet formulation	Saravanakumar et al. (2020)
Gd-CS-OA/Ce6	Breast cancer	Demonstrated promising application *in situ* 4T1 tumor model	Zhao et al. (2020)

FA-CS, folic acid conjugated chitosan nanoparticle; GA-CS NPs, glycyrrhetinic acid-conjugated chitosan nanoparticle; HER2-Gem-CS, herceptin (HER2)-conjugated gemcitabine-loaded chitosan nanoparticle; CNPs-Ce6, Chlorin e6 chitosan nanoparticles; CS-TPP/IL-12, chitosan-tripolyphosphate interleukin-12; HMSNs-CS-DOX-CuS, hollow mesoporous silica nanoparticles chitosan doxorubicin and copper sulfide; Gd-CS-OA/Ce6, gadopentetic acid chitosan octadecanoic acid/chlorin e6.

#### 4.2.2 Effects of Chitosan and its Derivatives on Innate Immune Response in Cancer Therapy

Chitosan may activate dendritic cells (DCs) to enhance the antitumor activity of natural killer (NK) cells by upregulating IFN-γ production. Moreover, it is reported that chitosan enhances NK cell activity to kill leukemia cells (Li et al., 2018). Chitosan has also been used as a vaccine adjuvant for regulating tumor microenvironment (TME). The TME plays a vital role in cancer control and elimination (Katsuta et al., 2020). Currently, immune cell becomes a promising candidate for drug delivery and has very broad spectrum tumor-targeting properties. However, the off-target problems and effective release strategies stand in front of us when using immune cells as drug carrier. It is reported that co-delivery of chitosan and IL-12 resulted in high inhibition of tumor growth (Yang and Zaharoff, 2013; Eslahi et al., 2021). Additionally, chitosan based nanoparticle is a potential delivery system for DNA vaccine and IL-12 is an effective gene adjuvant, which can induce strong antitumor immune response. It is reported that chitosan suspension or nanoparticles have immunostimulatory activities and inhibit tumor growth by inducing polarization of macrophage to enhance antitumor activity (Nandgude and Pagar, 2021). Taking chitosan solution as an adjuvant intervention for patients with lung cancer can improve immunity during radiotherapy (Ma et al., 2015). Chitosan also shows a biological activity for activating macrophages for tumoricidal activity and for production of interleukin-2 (Yang et al., 2019b; Lima et al., 2021).

#### 4.2.3 The Directed Effects of Chitosan and its Derivatives on Cancer Progression

Chitosan is a relatively low toxic and compatible polysaccharide. It is reported that administration of chitosan or oligochitosans can shield body from cancer induced oxidative stress. While, their anti-metastatic activity is mainly due to enhancing penetration properties (Kadry et al., 2018; Amirani et al., 2020). The migration of MDA-MB-231 human breast carcinoma cells weakened with elevated concentration of chitosan (Nam and Shon, 2009). Increasing the biodistribution of drugs is another mechanism for anticancer functionality of chitosan. The accumulation of the drug in tumor cells is due to chitosan improving cell permeability and drug retention time to low toxicity (Adhikari and Yadav, 2018).

Self-assembled microparticles from chitosan (SAMC) shows anticancer effect in human breast cancer cell lines and exhibits tumor growth inhibition in Ehrlich ascites tumor (EAT) bearing mice model. What’s more, SAMC decreases VEGF secretion in ascites, which is accompanied with reduction in neovessel formation. Consequently, SAMC may be another potential antitumor dietary supplement (Punarvasu and Prashanth, 2022). Modification of chitosan through its amino, acetamido, and hydroxy groups can give a veriety of derivatives with enhancing solubility and remarkable anticancer activity. Both chitosan and its various derivatives have been reported to show anticancer activity involving different cellular apoptotic pathways (Jiang et al., 2011; Jiang et al., 2015).

#### 4.2.4 Effects of Chitosan and its Derivatives on the Signal Pathway in Cancer Therapy

Phosphatidylinositol 3-kinase (PI3K)-AKT pathway is an important kinase signaling networks in carcinogenesis. The abnormal activation of PI3K-AKT signaling pathway has been implicated in numerous malignancies including endometrial, hepatocellular, breast, colorectal, prostate and cervical cancer (Franke, 2008; Amirani et al., 2020). Thus regulation and blockage of this kinase and its key molecules may be a potential approach in cancer therapy, and tremendous efforts have been made to achieve this goal. It is reported that chitosan and its derivatives down-regulate AKT phosphorylation in a dose-dependent manner and being used to block AKT activities in different cancer types (Liu et al., 2011; Xiong et al., 2018; Amirani et al., 2020; Fang et al., 2021).

Additionally, chitosan could induce apoptosis through increasing the concentration of calcium ion, level of ROS and mitochondrial membrane potential. Too many Fas/FasL pathway associated proteins were expressed abnormally in cancer (Gao et al., 2020). Chitosan may regulate the expression of apoptosis associated proteins, subsequently activated the cleavage of caspase-9 and caspase-3, which finally induced apoptosis in mitochondrial pathway (Wu et al., 2018; Wu et al., 2020).

Furthermore, chitosan oligosaccharide may inhibit the abnormally up-regulated programmed cell death ligand 1 (PD-L1) after chemotherapy in various tumors via the MAPK activation and STAT1 inhibition to improve efficacy of T cell mediated immune killing in tumors, which indicates that chitosan oligosaccharide may be used to improve the efficacy of existing chemotherapies (Chen et al., 2022).

## 5 Conclusion and Perspectives

Cancer is a major contributor to the global disease burden and is likely to continue in the next 20 years. Though great efforts have been made on cancer treatment in the past decades, cancer associated motality is still the leading cause of death. Intensive studies on chitosan have made it to be one of the most important and potential polymers for cancer treatment. Chitosan-based nanoparticles have exhibited exciting antitumor efficacy both *in vitro* and *in vivo*, which indicates that there is vast scope of clinical application. The favorable properties of chitosan have made it an ideal nanocarrier for drug controlled-release. Chitosan-based vehicle can be used for the encapsulation or conjugation of chemotherpeutic drugs, therapeutic gene nucleic acids, photosensitizers and cytokines and so on, achieving a more reliable targeted therapy for cancer. What’s more, chitosan itself also exhibits inhibitory effects on tumor cells through different signal pathways.

However, the clinical use of chitosan still confronts with some major obstacles. First, few studies have tested the safety of chitosan *in vivo*. Secondly, the chemical versatility of chitosan makes it more difficult to identify each entity. Last but not the least, it is unclear whether the nanoparticle targeting mechanism shown in rodent tumor models works the same in cancer patients. Consequently, the clinical application of antitumor chitosan nanoparticles should still be done with much caution, and an in-depth further clinical translation studies are urgently needed.
